# Investigation of an Imported Case of Rabies in a Juvenile Dog with Atypical Presentation

**DOI:** 10.3390/ani1040402

**Published:** 2011-11-18

**Authors:** Nicholas Johnson, Alex Nunez, Denise A. Marston, Graeme Harkess, Katja Voller, Trudy Goddard, Daniel Hicks, Lorraine M. McElhinney, Anthony R. Fooks

**Affiliations:** 1 Wildlife Zoonoses and Vector-Borne Diseases Research Group, Animal Health and Veterinary Laboratories Agency, Woodham Lane, Surrey KT15 3NB, UK; E-Mails: Denise.Marston@ahvla.gsi.gov.uk (D.A.M.); Graeme.Harkess@ahvla.gsi.gov.uk (G.H.); Katja.Voller@ahvla.gsi.gov.uk (K.V.); Trudy.Goddard@ahvla.gsi.gov.uk (T.G.); Lorraine.McElhinney@ahvla.gsi.gov.uk (L.M.M.); Tony.Fooks@ahvla.gsi.gov.uk (A.R.F.); 2 Pathology and Host Susceptibility Department, Animal Health and Veterinary Laboratories Agency, Woodham Lane, Surrey KT15 3NB, UK; E-Mails: Alejandro.Nunez@ahvla.gsi.gov.uk (A.N.); Daniel.Hicks@ahvla.gsi.gov.uk (D.H.); 3 National Consortium for Zoonosis Research, University of Liverpool, Leahurst, Chester High Road, Neston CH64 7TE, UK

**Keywords:** rabies virus, dog, quarantine, Sri Lanka

## Abstract

**Simple Summary:**

This study confirms the need for vigilance and rapid diagnosis of veterinary samples to control the unexpected importation of rabies into a country, particularly when the disease signs are atypical.

**Abstract:**

Movement of dogs between rabies-endemic and rabies-free countries carries the inherent risk of introducing the disease. In April of 2008, a juvenile dog was imported to the UK from Sri Lanka. It died shortly after transfer to a quarantine facility in the south-east of England following a short history of diarrhoea and convulsions but no overt signs of aggression. Subsequent investigation confirmed that rabies was the cause of death. Rabies virus was isolated from brain samples taken from the dog and the subsequent phylogenetic investigation confirmed that the genomic sequence from this virus shared over 99% homology with endemic rabies viruses from Sri Lanka. Histological examination of the brain demonstrated clear signs of encephalitis and rabies antigenic labeling in numerous neurons. In this particular case, Negri bodies were absent. As this case was diagnosed in a quarantine facility, the ‘rabies-free’ status of the UK was un-affected.

## 1. Introduction

The domestic dog is the most significant reservoir for rabies virus and dog bites are responsible for the great majority of human deaths from rabies [1]. Although rabies in dogs has been controlled and eliminated in many regions of the world, it still persists throughout Africa and Asia causing thousands of human deaths every year. Despite the availability of effective vaccination, it is only through control of dog rabies that human deaths due to rabies will be reduced [2]. The United Kingdom (UK) has been rabies free since 1922 [3]. The gradual reduction of rabies throughout western Europe, where sylvatic rabies had been endemic since the late 1960s, has further reduced the risk of rabies entering the country from mainland Europe. A similar situation now exists in much of Europe where the introduction of oral-vaccination campaigns has been highly effective at eliminating fox rabies [4]. However, the danger of importation is always present and there have been a number of well documented cases of rabies introduction, particularly through movement of dogs from Morocco into Spain and France [5]. For the UK, two methods of preventing rabies entry have been in use over the past decade. The first has been quarantine of companion animals and exotic species, particularly bats and carnivores. Figures for the numbers of dogs and cats entering quarantine between 1999 and 2005 are shown in Table 1. A decline in the number of animals entering quarantine was observed as a result of the introduction in 2000 of the second method of controlling entry of companion animals, the UK Pet Travel Scheme (PETS). This enabled immediate entry for dogs, cats and ferrets provided the animal complies with the requirements of the scheme, principally demonstration of vaccination against rabies. This scheme has proven highly popular with companion animal owners with approximately 750,000 animals entering the UK by this route between 2000 and 2010 (Table 2). Both these mechanisms of companion animal entry into the UK will be harmonized with those of other European Union member states at the start of 2012 [6].

Meldrum (1988) [7] reported 29 cases of rabies in quarantine animals up to 1988. On two occasions, cases of rabies have resulted in dogs after the six-month quarantine period [8] and as a consequence compulsory vaccination was introduced in 1974 for all dogs and cats entering quarantine. In both cases, the incursions were dealt with swiftly and no further incidence of the disease was reported. To date, the UK has never resorted to the use of vaccination to control the disease in wildlife although this contingency would be considered by the Department for Environment, Food and Rural Affairs (Defra), the government department responsible for animal disease control. Prior to 2008, the most recent rabies associated death in quarantine occurred in a dog imported from Zambia in 1990. There is a legal obligation to submit the head of any animal that dies in quarantine for rabies diagnosis. In 2008, there were 29 deaths in UK quarantine.

Two preliminary reports have previously described the clinical history of the case, rabies diagnosis [9] and the public health investigation that occurred [10]. Briefly, the dog was imported from Sri Lanka to the UK by an animal welfare charity. The dog arrived at Heathrow airport on the 17 April 2008, and spent one night at the Animal Reception Centre before being transferred to quarantine kennels in north-east London. The dog had a history of severe diarrhoea and convulsed violently before it died on the morning of the 25 April. The initial diagnosis was for infection with *Hepatozoon canis* for which it had been treated with doxycycline. Confirmation of rabies infection was made within 24 hours of the death of the dog. A further four dogs that had contact with the dog underwent euthanasia as a precautionary measure. Eleven individuals in the UK received post-exposure prophylaxis (PEP) following direct physical contact with the dog.

This study confirms the epidemiological link between this case of rabies and the epidemic of dog rabies in Sri Lanka. We also report the histological findings in brain sections of this animal and note the absence of Negri bodies in this particular case.

## 2. Materials and Methods

### 2.1. Rabies Virus Diagnosis

Rabies virus infection was confirmed on acetone fixed impression slides of brain samples using the Fluorescent Antibody Test [11] using FITC labeled anti-rabies monoclonal globulin (Fujirebio). This was confirmed using real-time RT-PCR [12] and virus isolation using the rabies tissue culture infection test (RTCIT) as previously described [13]. The mouse inoculation test was used following published guidelines [14]. Briefly, mice (n = 3) were inoculated with 30 μL homogenates prepared from brain samples and monitored for 28 days for the development of rabies.

### 2.2. Phylogenetic Analysis

RNA was extracted from brain samples using the TriZol™ method following the manufacturers protocols (Invitrogen). Total RNA was resuspended in HPLC grade water and diluted to 1 μg/μL. The primer Jw12 (5′-ATGTAACACC[C/T]CTACAATG-3′) was used to generate cDNA by reverse transcription with MMLV reverse transcriptase (Promega). The cDNA generated was used to amplify a 606 base pair (bp) fragment of the RABV nucleoprotein with primers Jw12 and Jw6dpl (5′-CAATTCGCACACATTTTGTG-3′). These primers were also used to sequence the fragment generated. Sequence analysis was conducted as described in Johnson *et al.* (2002) [15]. Phylogenetic analysis was undertaken on a 400 bp sequence of the RABV nucleoprotein gene (genome positions 71 to 470, based on the Pasteur virus genome, GenBank Accession number NC_001542) using the PHYLIP 3.5 package. One thousand bootstrap replicates were undertaken with values over 700 being considered significant. The consensus phylogenetic tree was visualised using the Treeview program, version 3.2.

### 2.3. Histopathology

Samples of the brainstem, hippocampus and cerebellum were dissected from the dog's brain and immediately fixed in formalin for five days. Fixed tissues were blocked and processed to paraffin wax. Serial 4 μm wax sections were cut and stained with hematoxylin and eosin (H & E) for histological examination or used for immunohistochemistry. Rabies virus nucleocapsid was detected using the monoclonal antibody HAM 5DF123B0 (a gift from the Swiss Rabies Centre, Switzerland). Antibody binding was detected with a biotinylated anti-mouse secondary antibody (Vector Laboratories) and amplified using an avidin-biotin-peroxidase conjugate with 3,3-diaminobenzidine (Sigma-Aldrich) for visualization, as previously described [16].

## 3. Results

**Figure 1:**
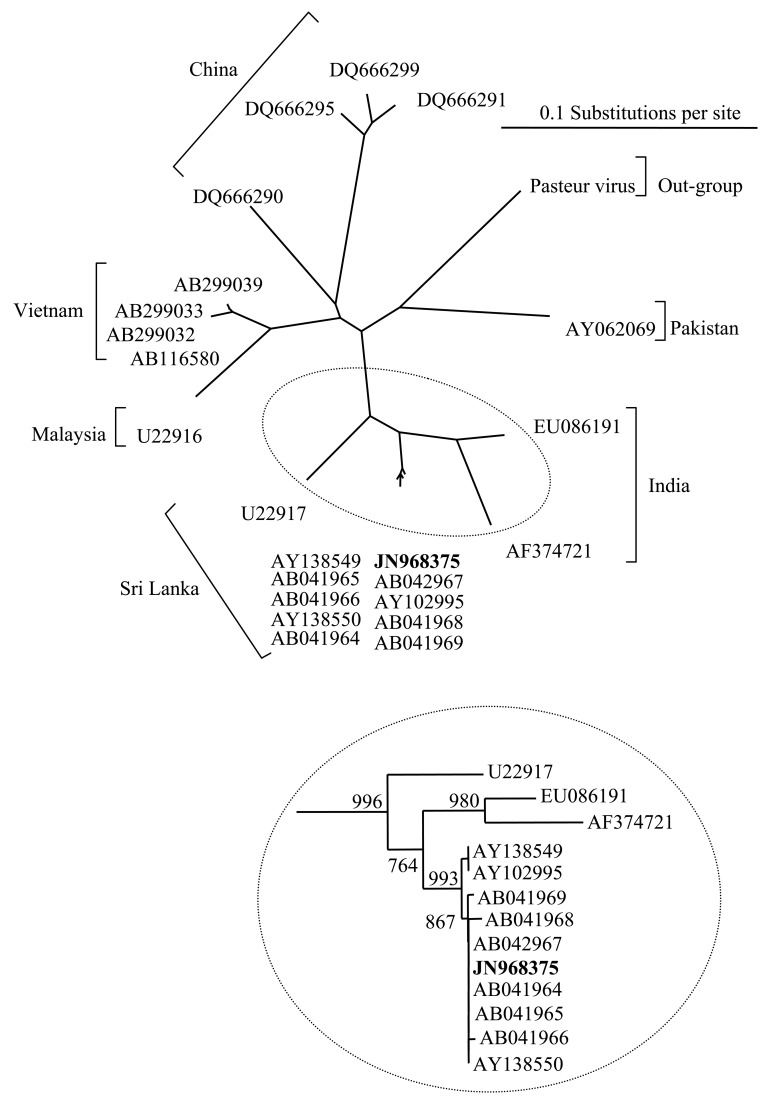
Phylogenetic analysis of rabies virus nucleoprotein sequences (400 bp) of isolates obtained from Asia. The Pasteur virus has been used as an out-group. The sequence from the UK isolate (RV2417) imported from Sri Lanka is shown in bold. Inset shows only isolate sequences from India and Sri Lanka.

Rabies virus infection was confirmed by the fluorescent antibody test and was corroborated by amplification of a genotype 1 lyssavirus by a differential reverse transcriptase-Polymerase Chain Reaction (RT-PCR) TaqMan assay from RNA extracted from the brain. Rabies virus was isolated from brain homogenates prepared directly from the animal and 100% of mice inoculated with this homogenate developed rabies encephalitis on day nine post-inoculation. A nested RT-PCR amplified a 606 bp fragment of the rabies nucleoprotein gene and this was used to derive 400 bp of genomic sequence from the infecting virus. Sequence alignment with a panel of isolates from Asia, and particularly from published sequences from Sri Lanka (Table 3), revealed that the RABV isolated from the dog had >99% sequence identity with RABV isolates from Sri Lanka. The phylogenetic tree (Figure 1 and inset) demonstrated that the isolated virus sequence (JN968375) clustered closely with a lineage of viruses isolated from India and the south-western provinces of Sri Lanka. This supports the evidence provided in the case history.

**Figure 2:**
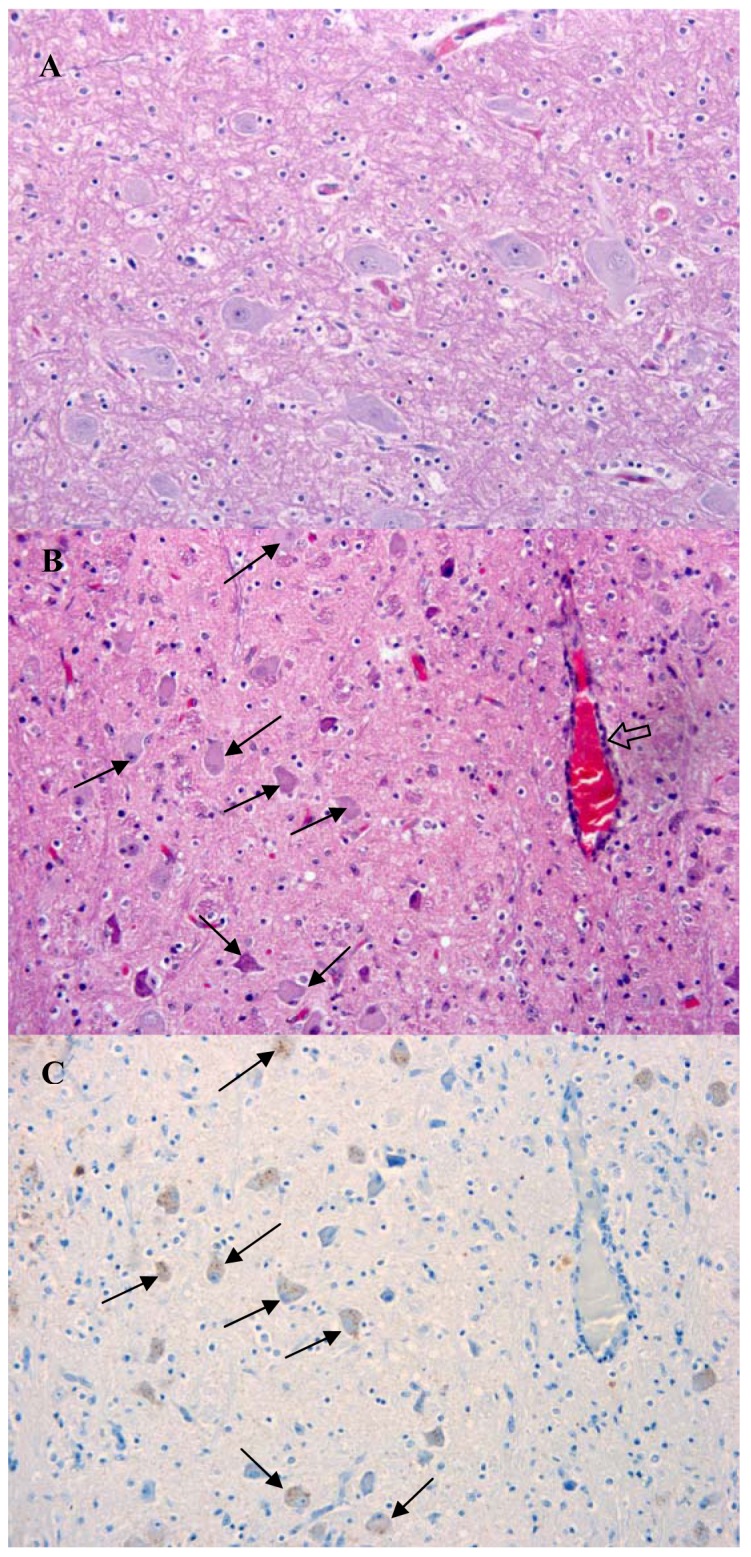
Pathological changes associated with rabies infection in the brain of the dog. (**A**) H & E staining of the brainstem of a normal dog. (**B**) H & E staining in the rabies positive dog. Arrows indicate central chromatolysis and nuclear margination. The block arrow indicates a perivascular cuff. (**C**) Immunohistochemistry labeling for RABV nucleoprotein as described in material and methods. Brown staining (arrows) indicate detection of RABV within neurons. Both images were taken at a magnification of 20×.

The presentation of brain sections revealed a mild non-suppurative encephalitis and leptomeningitis consistent with viral encephalitis. The characteristic changes were observed in all regions of the brain examined, including hippocampus, brainstem and cerebellum. They consisted of neuronal degeneration and necrosis, with occasional neuronal vacuolation, shrunken hyperchromatic neurons and images of central chromatolysis and nuclear margination, satellitosis, focal gliosis and lymphohistiocytic perivascular cuffing of no more than one cell of thickness [Figure 2(B)]. Inflammatory changes were more prominent in the brainstem. Negri bodies were not observed in any of the samples examined.

**Figure 3:**
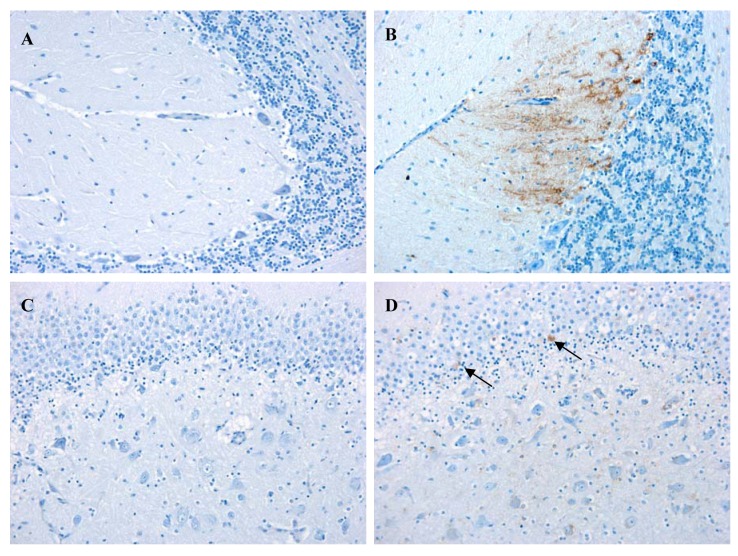
Staining for rabies virus nucleoprotein antigen in the cerebellum (**A & B**) and hippocampus (**C & D**) of a normal dog (**A & C**) and the RABV infected dog (**B & D**). Immunohistochemical detection (brown staining) was observed in Purkinje cells (perikaryon and cell processes) and in a few ganular cell layer neurons (**B**). A small number of immunolabelled neurons (arrows) are present in the hippocampus (**D**). All images were taken at a magnification of 20×.

Immunolabelling against rabies nucleocapsid was detected in the perikaryon and neuropil of neurons in all examined regions [Figure 2(C) and Figure 3]. No staining was observed in the corresponding regions of a normal dog brain used as a control [Figure 2(A) and Figure 3(A)]. The region with a higher number of immunolabelled neurons and with a more prominent immunolabelling in their perikaryon was the brainstem (Figure 2). Immunolabelling in the hippocampus was observed in a small number of pyramidal neurons [Figure 3(D)]. Detection of viral antigen in the cerebellum was occasional in the Purkinje cell layer [Figure 3(B)] and more frequent in the cells of the granular and molecular layer.

## 4. Discussion

Quarantine of dogs entering the UK has been an effective measure for preventing the entry of rabies since the beginning of the 20th century. Despite the introduction of the UK and EU PETS, which caused a noticeable drop in the number of animals entering quarantine, this has been an option for those wishing to import dogs into the country with over 3,000 entries in 2005 (Table 2). Despite the introduction of vaccination either in the country of origin or upon entry into quarantine, the risk still exists that an animal may have become infected during the period prior to entering the UK and that infection has advanced to a point where vaccination becomes ineffective. A further factor that may reduce the effectiveness of vaccination is the age of the dog [24]. Two recent cases of rabies in juvenile dogs in North America have highlighted this issue [25,26]. Vaccine manufacturers do not recommend rabies vaccination of dogs before twelve weeks of age due to possible presence of maternal antibodies that may inhibit the immune response [27]. This provides a window of susceptibility for those young dogs that do not have maternal antibody protection, as is likely in the case described here. Rabies is endemic in Sri Lanka with 68 cases of human rabies and 705 cases of dog rabies reported in 2006 [28]. However, these figures may be higher due to under reporting [29]. This has been in the face of large scale dog vaccination campaigns with approximately 1 million dogs vaccinated in 2006. A phylogenetic comparison of the sequence derived from the UK case with a panel of RABV sequences previously published by studies on the phylogeny of RABV from Sri Lanka [17,18] demonstrated a clear link between the case in quarantine and RABV circulating in Sri Lanka. These earlier studies have shown that the RABVs endemic in Sri Lanka are closely related to the viruses present in southern India. The Sri Lankan RABV isolated in the UK showed almost 100% sequence homology with some members of the panel and suggests that the dog was infected in the south-western provinces of Sri Lanka.

Although the disease symptoms observed in this animal were not typical of a rabies infected dog, investigation of samples by the FAT test demonstrated that the brain was heavily infected with RABV. This was confirmed by immunohistochemistry, which revealed widespread labelling of rabies nucleoprotein in the brainstem/medulla, hippocampus and, to a lesser extent, the cerebellum. The histological changes were consistent with viral encephalitis displaying characteristic degenerative and inflammatory changes. The nature and distribution of the lesions in this case were similar to those reported in a case of rabies in a 10-week-old puppy [25]. However, in the UK case and distinct from that observed in the Canadian dog, there was a complete absence of Negri bodies in all samples examined despite this being considered a common histological sign of rabies. This reflects the observation that up to 30% of street rabies cases do not produce Negri bodies [30,31] and emphasizes the requirement for virus-specific primary diagnostic tests such as the FAT and RT-PCR in cases of viral encephalitis of undetermined origin. The relatively high detection of viral antigen in the medulla/brainstem may be a consequence of the peripheral infection, as the medulla acts as crossing of motor tracts between the spinal cord and the brain. This in turn suggests that the brainstem should also be considered as a diagnostic sample when investigating animal cases suspected of rabies.

In March 2003 Defra launched DACTARI (Dog and Cat Travel and Risk Information). This is a national voluntary scheme for the investigation of the possible occurrence of exotic diseases in dogs and cats in Great Britain such as leishmaniasis, babesiosis, ehrlichiosis and dirofilariasis (of which leishmaniasis and ehrliciosis are zoonotic). Since 2003, there have been 100 reports of exotic diseases. Sixty-seven percent of which were detected in animals imported under PETS and 25% were detected in animals within quarantine.

## 4. Conclusions

Rabies virus is endemic in domestic dog populations in many regions of the world and as a result of movement of companion animals between countries the risk persists of transfer of infected animals between different areas, resulting in new outbreaks. Within rabies-free areas of the world, the consequences of such introductions are varied and range from a need to control the initial outbreak to investment in surveillance to demonstrate freedom from disease. For zoonotic viruses, there is a risk to public and veterinary health, which for rabies would result in human and animal deaths. Compulsory vaccination of dogs and restrictions on dog movement would also be required. In the case described in this study, no onward transmission occurred and a potential outbreak was controlled. However, the case demonstrates the need for constant vigilance by public health bodies in detecting incursions of potentially rabies infected animals and providing a rapid response to protect human contacts from this fatal disease.

## Figures and Tables

**Table 1:** Dogs and cats imported into the UK between 1999 through 2005. Data obtained from The Report of the Chief Veterinary Officer—Animal Health 2005 (Defra, UK).

**Year**	**Entering by quarantine**
1999	6,989 *
2000	5,296
2001	5,304
2002	3,555
2003	4,405
2004	3,514
2005	3,337

* The PETS scheme was introduced in 2000.

**Table 2:** Dogs, cats and ferrets entering the UK under the PETS Travel Scheme between 2000 through 2010 (Data published by Defra, UK).

**Year**	**Cats**	**Dogs**	**Ferrets**	**Annual Total**	**Cumulative Total**
2000	2,062	12,633	0	14,695	14,695
2001	3,562	23,158	0	26,720	41,415
2002	4,359	36,410	0	40,769	82,184
2003	6,012	48,938	0	54,951	137,134
2004	7,314	57,418	10	64,742	201,876
2005	8,346	69,531	39	77,916	279,792
2006	8,375	74,403	32	82,810	362,602
2007	10,137	89,127	43	99,307	461,909
2008	10,287	93,719	52	104,058	565,967
2009	7,128	89,376	55	96,559	662,526
2010	7,105	78,076	56	85,237	747,736
Total	7,4687	672,789	287	747,763	

**Table 3:** Details of rabies virus isolates used in the phylogenetic analysis of the quarantine case.

**Isolate reference No.**	**Country of origin**	**Host**	**Genbank reference**	**Reference**
RV2417	UK (ex Sri Lanka)	Dog	JN968375	This study
SRL1032	Sri Lanka	Jackal	AB041964	[17]
SRL1036	Sri Lanka	Human	AB041965	[17]
SRL1060	Sri Lanka	Dog	AB041966	[17]
SRL1077	Sri Lanka	Mongoose	AB041967	[17]
SRL1143	Sri Lanka	Cat	AB041968	[17]
SRL1145	Sri Lanka	Water Buffalo	AB041969	[17]
RV69	Sri Lanka	Dog	AY102995	[15]
1294	Sri Lanka	Dog	AY138549	[18]
5657	Sri Lanka	Bovine	AY138550	[18]
?	India		AF374721	[19]
9702INDI	India	Human	EU086191	[20]
94257SRI	Sri Lanka	Human	U22917	[21]
HCM2	Vietnam	Dog	AB299033	Unpublished
HCM10	Vietnam	Dog	AB299039	Unpublished
HCM1	Vietnam	Dog	AB299032	Unpublished
VN52	Vietnam	Dog	AB116580	Unpublished
8677MAL	Malaysia	?	U22916	[21]
Guizhou A148	China	Dog	DQ666291	[22]
Henan Sq9	China	Dog	DQ666299	[22]
Guizhou Qx2	China	Dog	DQ666295	[22]
Guizhou A103	China	Dog	DQ666290	[22]
RV277	Pakistan	Goat	AY062069	[15]
Pasteur virus	-	-	M13215	[23]

## Declaration

© 2011 Crown Copyright. This is under the terms of the Open Government Licence which governs the use and publication of all Crown copyright material. For more information on the terms of the Licence and how the contribution should be credited please follow this link http://www.nationalarchives.gov.uk/information-management/uk-gov-licensing-framework.htm.
